# Adipogenic RNAs are transferred in osteoblasts via bone marrow adipocytes-derived extracellular vesicles (EVs)

**DOI:** 10.1186/s12860-015-0057-5

**Published:** 2015-03-18

**Authors:** Perrine J Martin, Nathalie Haren, Olfa Ghali, Aline Clabaut, Christophe Chauveau, Pierre Hardouin, Odile Broux

**Affiliations:** Univ Lille Nord de France, F-59000 Lille, France; PMOI EA 4490, IFR 114, F-62327 Boulogne sur Mer and F-59000, Lille, France; Université du Littoral Côte d’Opale, ULCO, F-62327 Boulogne sur Mer, France; UCEIV EA 4492, F-59140 Dunkerque, France; Université du Littoral Côte d’Opale, EA4492 - Unité de Chimie Environnementale et Interactions sur le Vivant (UCEIV), Maison de la Recherche en Environnement Industriel 2, ULCO, 189A, Avenue Maurice Schumann, 59140 Dunkerque, France

**Keywords:** Extracellular vesicles, RNA transfer, Adipocytes, Osteoblasts, Osteoporosis

## Abstract

**Background:**

In osteoporosis, bone loss is accompanied by increased marrow adiposity. Given their proximity in the bone marrow and their shared origin, a dialogue between adipocytes and osteoblasts could be a factor in the competition between human Mesenchymal Stem Cells (hMSC) differentiation routes, leading to adipocyte differentiation at the expense of osteoblast differentiation. The adipocyte/osteoblast balance is highly regulated at the level of gene transcription. In our work, we focused on PPARgamma, CEBPalpha and CEBPdelta, as these transcription factors are seen as master regulators of adipogenesis and expressed precociously, and on leptin and adiponectin, considered as adipocyte marker genes. In 2010, our group has demonstrated, thanks to a coculture model, that in the presence of hMSC-derived adipocytes (hMSC-Adi), hMSC-derived osteoblasts (hMSC-Ost) express lesser amounts of osteogenic markers but exhibit the expression of typical adipogenic genes. Nevertheless, the mechanisms underlying this modulation of gene expression are not clarified. Recently, adipocytes were described as releasing extracellular vesicles (EVs), containing and transferring adipocyte specific transcripts, like PPARgamma, leptin and adiponectin. Here, we investigated whether EVs could be the way in which adipocytes transfer adipogenic RNAs in our coculture model.

**Results:**

We observed in hMSC-Ost incubated in hAdi-CM an increase in the adipogenic PPARγ, leptin, CEBPα and CEBPδ transcripts as well as the anti-osteoblastic miR-138, miR30c, miR125a, miR-125b, miR-31 miRNAs, probably implicated in the observed osteocalcin (OC) and osteopontin (OP) expression decrease. Moreover, EVs were isolated from conditioned media collected from cultures of hMSC at different stages of adipocyte differentiation and these specific adipogenic transcripts were detected inside. Finally, thanks to interspecies conditioned media exposition, we could highlight for the first time a horizontal transfer of adipogenic transcripts from medullary adipocytes to osteoblasts.

**Conclusions:**

Here, we have shown, for the first time, RNA transfer between hMSC-derived adipocytes and osteoblasts through EVs. Additional studies are needed to clarify if this mechanism has a role in the adipocytic switch driven on osteoblasts by adipocytes inside bone marrow and if EVs could be a target component to regulate the competition between osteoblasts and adipocytes in the prevention or in the therapy of osteoporosis and other osteopenia.

## Background

Maintenance of healthy bone mass requires a continuous process of bone renewal, known as bone remodelling, which consists of a balance of bone resorption by osteoclasts and bone formation by osteoblasts. The decrease in bone mass that occurs in osteoporosis results from the impairment of this balance, leading to increased fracture risk. Additional evidence from *in vitro*, *in vivo* and clinical studies also supports a link between bone loss and accumulation of medullary adipocytes [1-5]. Situations such as aging, estrogen insufficiency [6], anorexia nervosa [7], diverse therapies [8], microgravity exposure [9], or factors such as miRNAs [10,11] are known to induce bone loss concurrently with enhanced bone marrow adiposity. Evidence shows that a dialogue between adipocytes and osteoblasts is one of the mechanisms occurring in the competition between human Mesenchymal Stem Cells (hMSC) differentiation routes, supporting adipocyte differentiation at the expense of osteoblast differentiation. Consequently, factors which promote adipogenesis not only lead to “fatty marrow” but also inhibit osteoblastogenesis or osteoblast proliferation, resulting in decreased osteoblast numbers, diminished bone formation and, potentially, loss of bone mass leading to osteoporosis [12,13]. The adipocyte/osteoblast balance is highly regulated at the level of gene transcription [14]. It is difficult to find specific adipogenic mRNAs that are not shared by osteoblasts [15,16]. In our work, we focused on one side on PPARγ, CEBPα and CEBPδ, as these transcription factors are seen as the master regulators of adipogenesis [17] and are expressed precociously, and on the other side on leptin and adiponectin, considered as adipocyte marker genes increasing in a time-dependent manner during adipogenic induction [15].

In 2010, our group has demonstrated, using a coculture model [18], that in the presence of hMSC-derived adipocytes (hMSC-Adi), hMSC-derived osteoblasts (hMSC-Ost) express lower amounts of osteogenic markers but exhibit expression of typical adipogenic genes. Nevertheless, the mechanisms underlying this modulation of gene expression are not clarified. Extracellular Vesicles (EVs) are 100 nm to 1 μm membrane-bound structures released from the plasma membrane of most cell types and are involved in a range of physiological processes, including angiogenesis, inflammation, progression of cancers and reprogramming of mesenchymal stem cells, especially by transferring RNAs [19]. Recently, primary rat and cultured mouse adipocytes were described as releasing EVs [20]. They have been shown to contain adipocyte specific transcripts, like leptin and adiponectin, that are both transferred into and expressed in acceptor adipocytes and are involved in the upregulation of lipogenesis and cell size [21]. Moreover, adipocyte-derived vesicles were demonstrated to transfer adipocyte-specific gene transcripts such as adiponectin, resistin, and PPARγ2 into RAW264.7 macrophages [22].

Here, we considered if EVs could be the mechanism by which adipocytes transfer adipogenic RNAs in our coculture model. To confirm this hypothesis, we incubated hMSC-Ost in conditioned medium obtained from hMSC-Adi (hAdi-CM) cultures. We observed in the osteoblastic population an increase in the adipogenic PPARγ, leptin, CEBPα and CEBPδ transcripts, dependent on mRNA amount as shown by conditioned media obtained from adipocytes at several differentiation stages and PPARγ silencing experiments, as well as the anti-osteoblastic miR-138, miR30c, miR125a, miR-125b, miR-31 miRNAs [23-26], probably implicated in osteocalcin (OC) and osteopontin (OP) expression decrease. Moreover, we isolated EVs from conditioned media collected from cultures of hMSC at different stages of adipocyte differentiation and detected inside the presence of these specific adipogenic transcripts. Finally, thanks to inter-species conditioned media exposition, and using species specific primers, we could highlight for the first time a horizontal transfer of adipogenic transcripts from medullary adipocytes to osteoblasts. Here, we postulate that hMSC-derived adipocytes are able to produce EVs responsible for the transfer of adipogenic RNAs to osteoblasts.

## Results

### Adipocyte-specific RNA expression is increased in osteoblasts by hMSC-derived adipocyte conditioned media and influence osteoblast markers expression

**Figure 1:**
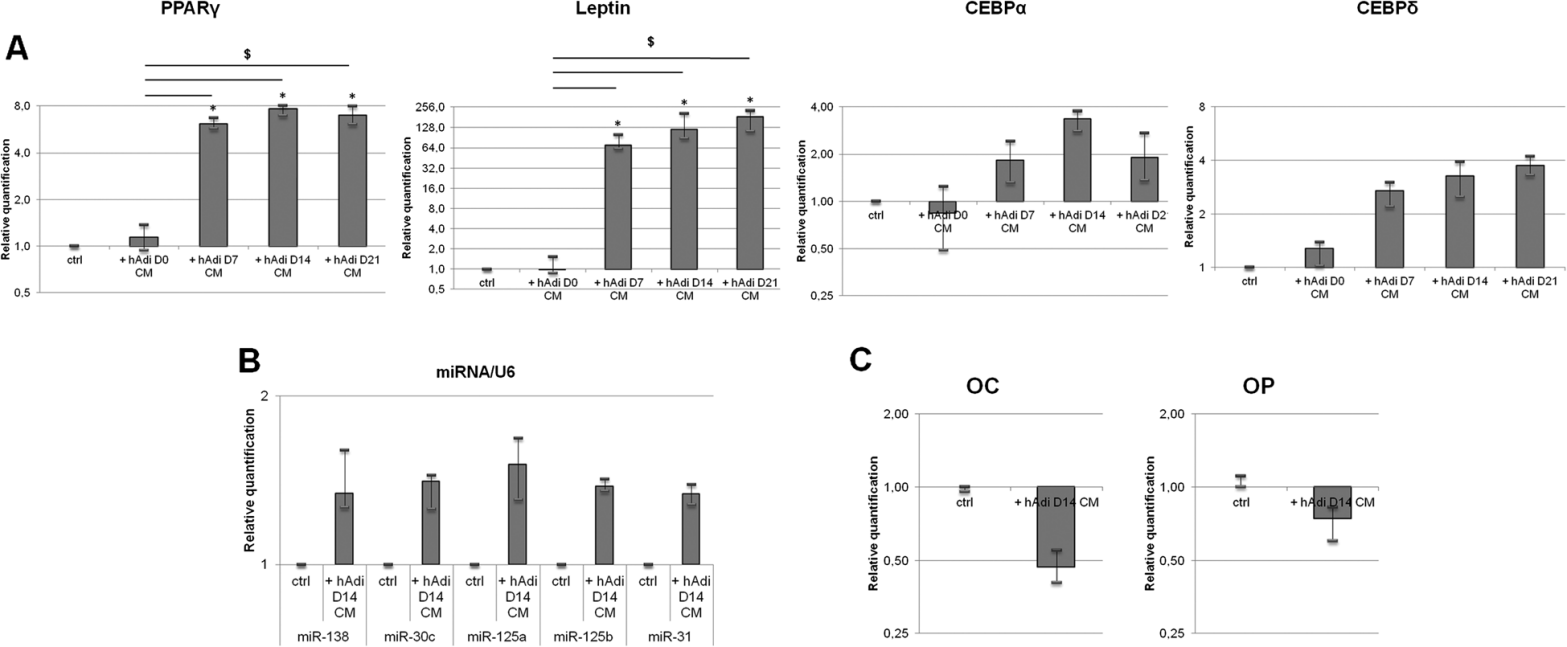
**Adipocyte- and osteoblast-specific mRNA and miRNA quantification in osteoblasts incubated with hMSC-0, 7, 14 and 21 days differentiated adipocyte medium. (A)** RT-qPCR analysis of PPARγ, Leptin, CEBPα and CEBPδ in 14 days-differentiated osteoblasts incubated with conditioned medium from 0, 7, 14, and 21 days-differentiated adipocytes (hAdi-CM). Transcript levels of YWHAZ were used for sample normalization [29]. Data was obtained from 4 independent experiments (Bars: median ± interquartile space). Differences were considered significant at p < 0.05 (*p < 0.05). **(B)** RT-qPCR analysis of miR-138, miR-30c, miR-125a, miR-125b, miR-31 in 14 days-differentiated osteoblasts incubated with conditioned medium from 14 days-differentiated adipocytes (hAdi-CM). Transcript levels of RNU6P were used for sample normalization. Data was obtained from 3 independent experiments (Bars: median ± interquartile space). **(C)** RT-qPCR analysis of OC and OP in 14 days-differentiated osteoblasts incubated with conditioned medium from 0 (ctrl) or 14 days-differentiated adipocytes (hAdi D14-CM). Transcript levels of YWHAZ were used for sample normalization. Data was obtained from 3 independent experiments (Bars: median ± interquartile space). For **(A)**, **(B)** and **(C)** control (ctrl) was performed by incubating hMSC-derived osteoblasts with serum- and inductors-free DMEM for the same time.

hMSC-Ost and hMSC-Adi were obtained as described in Materials and Methods. We examined if hMSC-Ost incubation during 48 h in conditioned media issued from hMSC-Adi (hAdi-CM) after 0, 7, 14 and 21 days of differentiation, lead to a variation of adipocyte and osteoblast specific gene expression in osteoblasts. First, we measured by RT-qPCR expression levels of the four adipocyte-specific genes PPARγ, leptin, CEBPα and CEBPδ and relative quantification was performed on the reference gene YWHAZ. As shown in Figure 1A, relative expression levels of the four genes were directly dependent on the stage of differentiation of the hMSC-Adi. The maximal increase was reached with the CM obtained after 14 days of adipocyte differentiation for all of the genes. For this reason, miRNA and osteoblasts mRNA levels were measured in hMSC-Ost incubated in conditioned medium from adipocytes differentiated for 14 days. Five miRNAs, miR-138, miR-30c, miR-125a, miR-125b and miR-31, were selected for their capacity to inhibit osteoblast gene expression [25-28]. As shown in Figure 1B, all of them showed an increase in their expression in hMSC-Ost incubated with 14 days differentiated hAdi-CM. Furthermore, incubation of hOst D14 with hAdi D14 CM led to a slight but reproducible decrease of two late osteoblastic differentiation markers of gene expression, osteocalcin (OC) and osteopontin (OP) (Figure 1C), supporting the idea that at least some of the transferred miRNAs are functional.

Taken together, these results indicate that some compounds are secreted by differentiated hMSC-Adi leading to an increase in specific pro-adipogenic or anti-osteoblastic RNAs in osteoblasts.

### hMSC-Adi express adipogenic mRNAs and anti-osteoblastic miRNAs proportionally as those found in hMSC-Ost incubated in hAdi-CM

**Figure 2:**
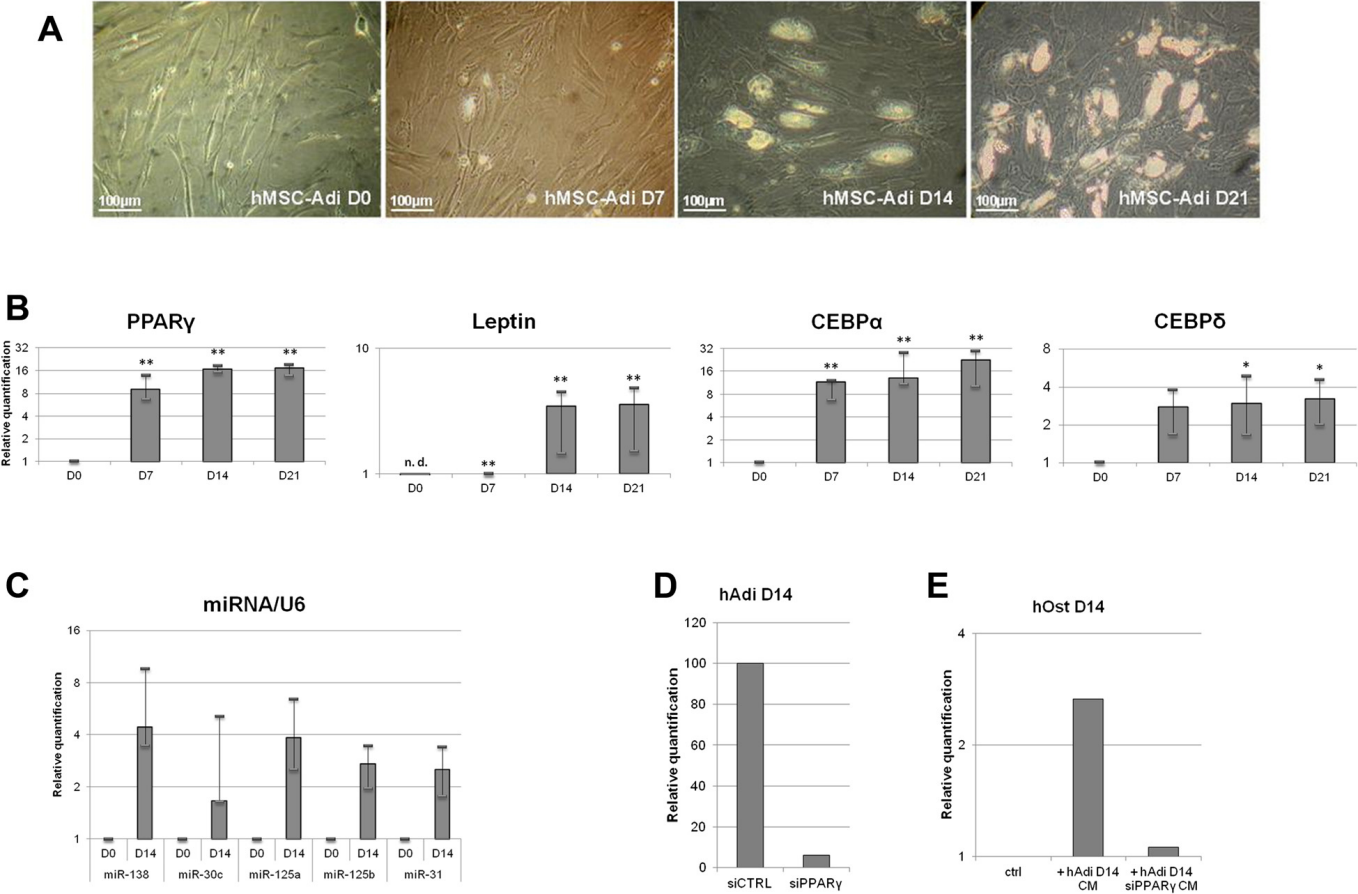
**Observation and adipogenic-specific RNAs quantification of adipocytes at 0, 7, 14 and 21 days of differentiation. (A)** Images obtained by optical microscopy on human MSC-derived adipocytes (hMSC-Adi) after 0, 7, 14, and 21 days of differentiation. Scale bars: 100 μm. **(B)** RT-qPCR analysis of PPARγ, Leptin, CEBPα and CEBPδ in 0, 7, 14, and 21 days-differentiated adipocytes. Transcript levels of GAPDH were used for sample normalization. Data was obtained from 4 independent experiments (Bars: median ± interquartile space). Differences were considered significant at p < 0.05 (*p < 0.05, **p < 0.01). (n. d. not detected). **(C)** RT-qPCR of miR-138, miR-30c, miR-125a, miR-125b, miR-31 in 0 and 14 days-differentiated adipocytes. Transcript levels of RNU6P were used for sample normalization. **(D)** RT-qPCR analysis of PPARγ in 14 days-differentiated adipocytes (hAdi D14) transfected with siPPARγ or with non-target control siRNA (siCTRL). Transcript levels of 36B4 were used for sample normalization. **(E)** RT-qPCR analysis of PPARγ in 14 days-differentiated osteoblasts incubated with conditioned medium from 0 (ctrl) or 14 days-differentiated adipocytes transfected (hAdi D14 siPPARγ-CM) or not (hAdi D14-CM). Transcript levels of 36B4 were used for sample normalization.

We wanted to confirm the increase in the same specific RNAs in hMSC-derived adipocytes at 0, 7, 14 and 21 days of differentiation as in osteoblasts incubated with the corresponding conditioned media. Figure 2A shows representative images of the differentiating hAdi used for the generation of CM. Appearance of lipid droplets after 7 days of differentiation whose number increases at 14 and 21 days were observed, as expected. In addition, Figure 2B shows that hMSC-Adi expressed increasing amounts of the four studied adipogenic mRNAs, depending on the stage of differentiation. More precisely, PPARγ, CEBPα and CEBPδ reached a maximal level of expression after 7 days of differentiation whereas the maximal level of leptin expression was reached after 14 days of differentiation, with no mRNA detected at day 0. Concerning the studied miRNAs, their expression was enhanced in 14 days differentiated hMSC-Adi (Figure 2C).

Furthermore, silencing experiments were performed to examine if knockdown of PPARγ mRNA in the donor adipocytes leads to a reduction of the corresponding RNA in the recipient osteoblasts after conditioned medium transfer. hAdi D14 were transfected with siRNA targeting PPARγ mRNA and a substantial decrease (more than 95%) could be obtained (Figure 2D). Incubation of hOst D14 with the subsequent conditioned medium (hAdi D14 siPPARγ CM) really led to a reduction of PPARγ mRNA in the recipient osteoblasts compared to those incubated with hAdi D14 CM (Figure 2E).

In conclusion, the expression of RNAs found in hMSC-Ost incubated with hAdi-CM follows the same tendency as those found in hMSC-Adi from which the CM is obtained, suggesting RNA transfer between the two cell types.

### Extracellular vesicles harbouring adipogenic mRNAs are found in hAdi-CM

**Figure 3:**
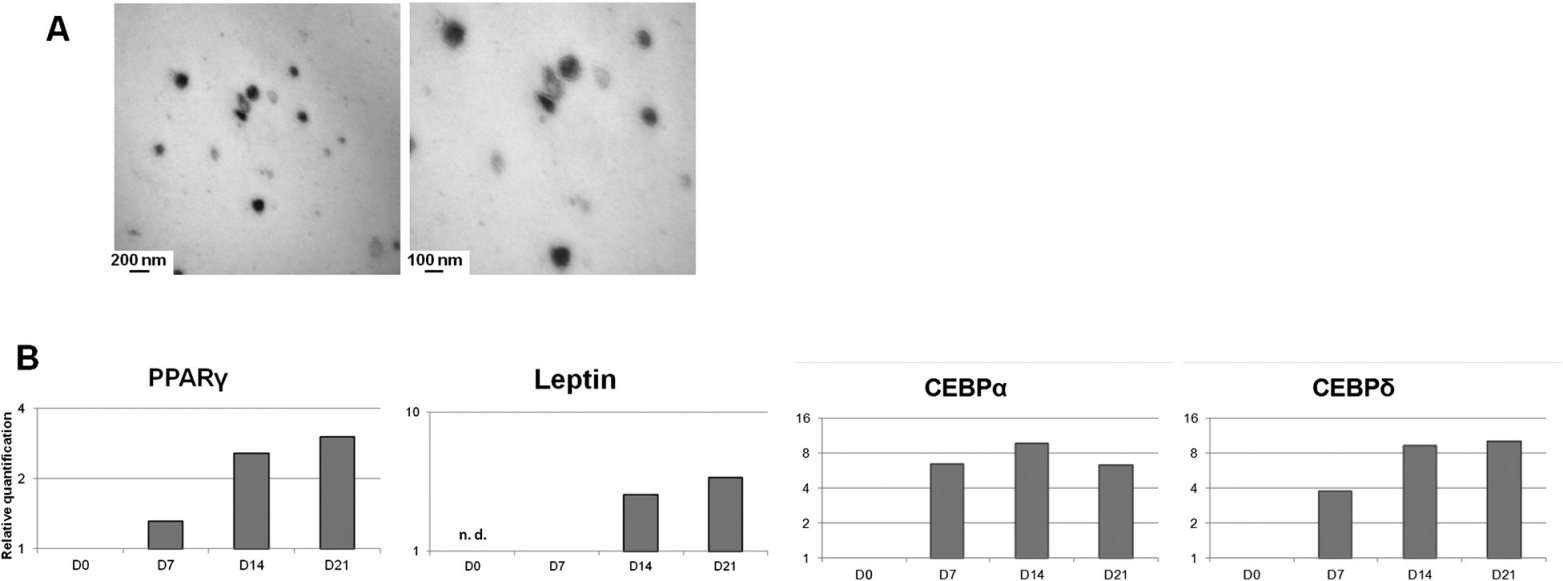
**Morphologic observation and adipocyte-specific mRNA quantification of EVs isolated from CM obtained from adipocytes after 14 days of differentiation. (A)** Images obtained by transmission electron microscopy on the EVs collected at day 14 of adipocyte differentiation of hMSC, after negative coloration by uranyl oxalate. Scale bars: 200 and 100 nm. **(B)** RT-qPCR analysis of PPARγ, Leptin, CEBPα and CEBPδ in EVs isolated from 0, 7, 14, and 21 days-differentiated adipocytes. Transcript levels of GAPDH were used for sample normalization.

We wanted to investigate if EVs can be one of the vectors taking part in the adipocyte gene rise described above. As a first confirmation, hMSC-Adi EVs were successfully isolated and purified from CM using the method of differential centrifugation and filtration [30]. The obtained hMSC-Adi EVs were observed under transmission electron microscopy, which revealed that they are approximately 30–100 nm in size (Figure 3A). RNAs were then isolated from these EVs at the differentiation times of their adipocytes of origin. As shown in Figure 3B, RT-qPCR experiments revealed that EVs hold as proportional amounts of specific mRNA as hMSC-Adi expressed depending on the differentiation stage. For example, no mRNA coding for leptin was detected in EVs isolated from the non-differentiated adipocyte (day 0) CM, as found in the cells of origin (Figure 2B, day 0). We also noticed the same maximum increase at day 7 of differentiation, maintained at day 14 and 21, of CEBPα and CEBPδ mRNAs in EVs as in hMSC-Adi. Finally, we observed a similar expression of each of these mRNAs in EVs and in hMSC-Ost incubated with hAdi-CM. These results suggest that EVs could be the vehicle by which adipogenic mRNAs are transferred to osteoblasts.

### Species-specific adipogenic mRNAs are transferred from adipocytes to osteoblasts

**Figure 4:**
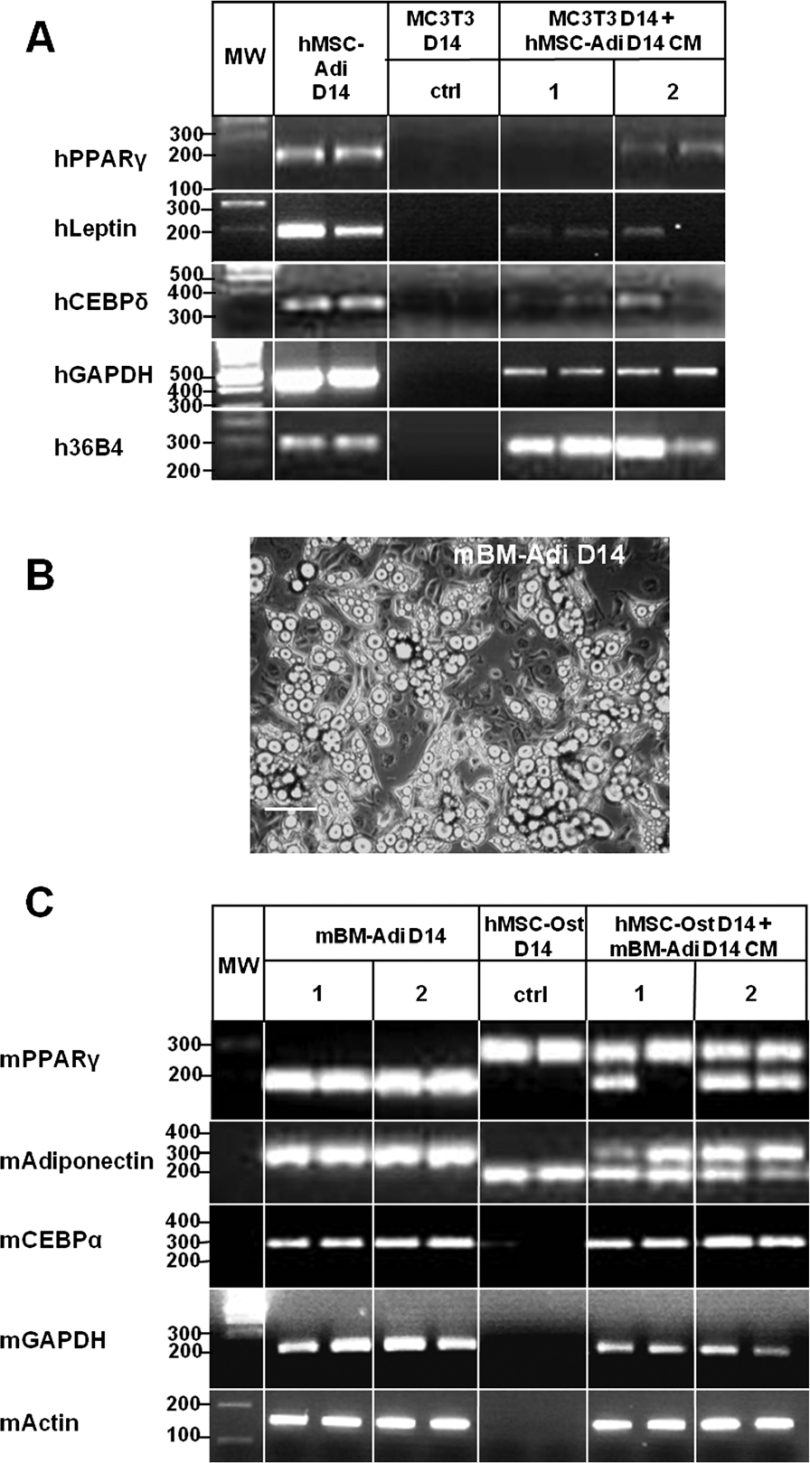
**Species-specific adipogenic mRNAs observation in osteoblasts incubated in different species adipocytes conditioned medium. (A)** Agarose gel electrophoresis of RT-PCR products of hPPARγ, hLeptin, hCEBPδ, hGAPDH and h36B4 from 14 days-differentiated MC-3 T3 (MC3T3 D14) incubated with conditioned medium from 14 days-differentiated human adipocytes (hMSC-Adi D14 CM). Two representative experiments are shown (1 and 2). Positive control was performed on hMSC-Adi D14 and negative control was obtained by incubating MC3T3 D14 with serum- and inductors-free DMEM for the same time (ctrl). **(B)** Images obtained by optical microscopy on mouse bone marrow-derived adipocytes (mBM-Adi) after 14 days of differentiation. Scale bar: 100 μm. **(C)** Agarose gel electrophoresis of RT-PCR products of mPPARγ, mAdiponectin, mCEBPα, mGAPDH and mActin from 14 days-differentiated human osteoblasts (hMSC-Ost D14) incubated with conditioned medium from 14 days-differentiated mouse adipocytes (mBM-Adi D14 CM). Two representative experiments are shown (1 and 2). Positive control was performed on mBM-Adi D14 and negative control was obtained by incubating hMSC-Ost D14 with serum- and inductors-free DMEM for the same time (ctrl).

To carry out inter-species experiments, hMSC-Adi CM was applied to the mouse cell line MC3T3-derived osteoblasts and RT-qPCR was performed with highly specific human primers against adipocyte specific genes. Each of these primers were tested on mouse RNA for their specificity (data not shown). hGAPDH and h36B4 genes, usually used as reference genes, were chosen as they are known to be commonly found in EVs [19]. As shown in Figure 4A, the human adipocyte genes hPPARγ, hLeptin and hCEBPδ were specifically amplified from RNA isolated from MC3T3. Similarly, hGAPDH and h36B4 RNAs were found to be amplified. These results confirm the hypothesis of a mRNA transfer from adipocytes to osteoblasts.

To strengthen these results, the reverse experiment was performed. Mouse bone marrow primary cells were obtained as described in Materials and Methods and were differentiated into adipocytes during 14 days (mBM-Adi D14). Observation by optical microscopy (Figure 4B) showed the appearance of lipid droplets confirming the adipocytic differentiation. Conditioned medium of mBM-Adi D14 (mBM-Adi D14 CM) was applied to hMSC-Ost and amplification of the mouse genes mPPARγ, mAdiponectin and mCEBPα was performed using highly specific primers as well (Figure 4C). Although a non-specific amplicon, especially for mPPARγ and mAdiponectin, appeared for hMSC-Ost D14, this happened in all conditions, confirming the non-specificity of the band. For both mRNA, a specific band at the right size is observed, only when hMSC-Ost D14 were incubated with mBM-Adi D14 CM. Moreover mBM-Adi D14 were used as a positive control for mouse genes amplification, showing a unique band at the corresponding size, confirming the specificity of the primers for the mouse PPARγ and Adiponectin mRNAs. mGAPDH and mActin genes were used as reference genes commonly found in EVs [19]. Taken together, these results highlighted that the three adipocyte specific mouse mRNAs mPPARγ, mAdiponectin and mCEBPα as well as mGAPDH and mActin were found in hMSC-Ost incubated with mBM-Adi D14 CM, supporting transfer of mRNAs from adipocyte derived from bone marrow primary cells.

## Discussion

The increase in marrow adipocytes observed in osteoporosis may contribute to limiting of osteoblast commitment by acting on MSCs or even directly on osteoblasts [31]. This hypothesis is supported by the proximity of adipocytes to osteoblasts in the bone marrow, their shared mesenchymal progenitor origin, and the fact that adipocytes are secretory cells. In an attempt to reproduce cellular interactions within the bone marrow, our group previously developed a coculture system using hMSC-derived osteoblasts and adipocytes [18]. In this model, a negative influence of adipocytes on osteoblast differentiation has been observed, supporting this assumption. Bone marrow adipocytes are highly secreting cells and, amongst factors such as fatty acids and adipokines already described *in vitro* to modify their neighbouring cells [32-34], we wonder if other mediators could be implicated. Within the past decade, EVs have emerged as important factors in intercellular communication, being involved in the transmission of biological signals between cells [19,35]. EVs components may include proteins, mRNAs and miRNAs. These components can be delivered to neighbouring cells and can be functional in their new location. The protein and RNA composition of EVs depends on the cell type or tissue source from which they originate. Although EVs are released from many cell types, to our knowledge no study has reported their secretion by hMSC-derived adipocytes.

In this study we could show that hMSC-Adi secrete EVs containing adipocyte specific mRNAs and anti-osteogenic miRNAs, that can be transferred to osteoblasts. Morphologically, the obtained EVs look quite homogeneous in size, around 200 nm, but their protein profile remains to be characterized to classify them either as exosomes or as ectosomes [36,37]. Both are membrane bound vesicles that differ based on their process of biogenesis, and biophysical properties, such as size and surface protein markers.

A significant increase of PPARγ, CEBPα and CEBPδ expression in osteoblasts incubated with hAdi-CM could be measured, suggesting an adipocyte-specific mRNA transfer. This hypothesis is corroborated by: (i) the presence of these transcripts in EVs, (ii) the fact that silencing of PPARγ mRNA in donor adipocytes inhibits the increase of PPARγ mRNA observed in the recipient osteoblasts after incubation in hAdi CM and (iii) the inter-species experiments, except for hCEBPα to mouse cells and mCEBPδ to human cells because of difficulties to optimize specific PCR. Besides, the genes used as controls, GAPDH, 36B4 and Actin, that are known to be found in EVs [19], are also transferred to their target cells (Figure 4A and C), corroborating the hypothesis of transcripts passage through EVs. Nonetheless, the bands observed in MC3T3 D14 incubated with hAdi D14 CM are slight and poorly reproducible, especially for hPPARγ and hLeptin. This can be explained by the fact that only a small quantity of human transcripts is transferred. For this reason, the observed amplification should be considered as qualitative more than quantitative. Concerning adiponectin, a time-dependent induction of its expression in hAdi could be measured, but no increase could be observed in human osteoblasts incubated with hAdi-CM (data not shown). However, a transfer of mouse adiponectin mRNA could be detected in hOst incubated with CM mBM-Adi by specific PCR.

An increase of miR-30c and miR-31 miRNAs, targeting osteogenic transcripts such as RUNX2 and Osterix, [23,26-28] and of miR-125a known to be significantly downregulated during osteogenic differentiation in human adipose-derived stem cells [38] and predicted to target the osteogenic genes Smad 2 and 4 [39], could be found in hOst incubated with hAdi-CM. However, no decrease of the expression of Runx2 and Osterix could be observed (data not shown) as described in studies using cells overexpressing these miRNAs [23,25,27,28]. In our case, the slight induction of each miRNA observed in hOst incubated with hAdi-CM is maybe not sufficient to reproduce a significant repression of Runx2 and Osterix. Nonetheless we could detect a small decrease of the late osteoblastic marker transcripts OC et OP. These were found to be targeted by miR-125b and miR-138 [23,40], two miRNAs that we have shown to be increased in hOst incubated with hAdi-CM, suggesting that the transferred miRNAs are functional. However their putative role in our model should be investigated in depth.

As RNA transfer between hMSC-derived adipocytes and hMSC-derived osteoblasts could be shown, we further examined whether EVs were directly implicated. hMSC-Ost were incubated with EVs isolated from hAdi-CM and no adipogenic RNA increase could be found in hMSC-Ost (data not shown). This result might be attributed to the loss of part of the EVs and/or of their functional effect during the isolation procedure. Another possible explanation is that EVs are not the only way in which adipocytes may influence osteoblast gene expression and maybe some adipocytes-secreted molecules are needed in the medium to enhance their effects. Further study is required to answer these questions.

## Conclusions

Here, we have shown for the first time that RNA transfer is possible between hMSC-derived adipocytes and osteoblasts through EVs. Additional studies are needed to clarify if this mechanism has a role in the adipocytic switch driven on osteoblasts by adipocytes inside bone marrow and if EVs could be a target component to regulate the competition between osteoblasts and adipocytes in the prevention or in the therapy of osteoporosis and other osteopenia.

## Methods

### Cell culture experiments

#### Human MSC culture and induction of osteogenic and adipogenic differentiation

Purified MSCs were purchased from Lonza (Verviers, Belgium) and were cultured as described previously [18]. Differentiation experiments were started when hMSCs had reached confluence (D0). To induce osteogenesis, hMSCs were cultured in DMEM with 10% FCS supplemented with osteogenic inductors (50 μM ascorbic acid, 10 mM β-glycerophosphate and 10^−8^ M vitamin D3 (Sigma-Aldrich Corporation, St Quentin Fallavier, France)) for 14 days (hMSC-Ost). For adipogenic differentiation, hMSCs were cultured in DMEM with 10% FCS supplemented with adipogenic inductors (0.5 μM dexamethasone, 0.5 mM isobutyl-1-methylxanthine and 50 μM indomethacin (Sigma-Aldrich Corporation)) for 0, 7, 14 or 21 days (hMSC-Adi).

#### MC3T3 culture and osteogenic differentiation

The MC3T3-E1 cells were plated in expansion medium composed of alpha-Modified Eagle Medium (α-MEM) (Dutscher) containing 10% FCS, 1% penicillin/streptomycin and 1% glutamine (Dutscher). Osteogenesis differentiation was started when MC3T3 had reached confluence (day 0) by incubation with osteogenic inductors (50 μg/ml ascorbic acid and 10 mM β-glycerophosphate (Sigma-Aldrich Corporation) for 14 days.

#### Primary mice bone marrow cell culture and adipogenic differentiation

Female C57BL6 mice, 7 weeks of age, were purchased from Charles River and were acclimatized for 1 week under standard laboratory conditions. Immediately after sacrifice, femurs and tibias were removed and cleaned of connective tissue, the ends were cut, and the marrow was flushed with α-MEM (PAN Biotech, Dutsher) supplemented with 15% FCS (PAN Biotech), 2 mM of glutamine (PAN Biotech), 50 IU/ml of penicillin (PAN Biotech), and 50 μg/ml of streptomycin (PAN Biotech). Single-cell suspensions were prepared in α-MEM by drawing the cells several times through graded needles. Cell density was determined using a Malassez counting chamber. For all experiments, cells were plated at a density of 2 × 10^6^ cells/cm^2^ and incubated at 37°C in 5% CO_2_. After 24 hours, non-adherent cells were removed and the medium was changed every 3 days until cell confluence.

Adipogenesis was induced by DMEM (PAN Biotech) 10% FCS supplemented with 10 μg/ml insulin/0,5 μM dexamethasone/100 μM indomethacin/500 μM 3-isobutyl-1-methylxanthine (Sigma-Aldrich Corporation) for 4 days and maintained in 10 μg/ml insulin/0,5 μM dexamethasone/5 μM pioglitazone (Sigma-Aldrich Corporation) for 10 days (mBM-Adi).

#### Conditioned medium (CM) experiments

After adipogenic differentiation of hMSCs or mouse bone marrow derived cells for the indicated times, differentiation medium was discarded and replaced with serum- and inductors-free DMEM containing antibiotics for 48 h and collected (hAdi-CM or mBM-Adi CM). 1 ml of CM was then applied on DMEM rinsed hMSC- or MC3T3-derived osteoblasts cultured in 12-well plates and incubated for 48 h. As controls, osteoblasts were incubated with serum- and inductors-free DMEM for the same time.

#### Small interfering RNA (siRNA) transfection

RNA interference was used to knockdown the expression of PPARγ in hMSC-Adi. siRNA for PPARγ (siPPARγ) and non-target controls were designed and synthesized by Dharmacon (ABgene Ltd, United Kingdom) and distributed by Thermo Fisher Scientific (PPARG Accell SMART pool, E-003436 and Accell control siRNA kit, K-005000). hAdi D14 were transfected with siRNAs (1 μM) for 72 h according to the manufacturer’s instructions.

hMSC-Ost were incubated 48 h in conditioned medium from hMSC-Adi transfected (hAdi D14 siPPARγ-CM) or not (hAdi-CM) with siPPARγ.

#### Extracellular vesicles (EVs) isolation

hMSC-derived adipocytes (hMSC-Adi) at the indicated times of differentiation (D0, D7, D14, D21) were incubated in EVs-free DMEM and the obtained conditioned medium from 1.5 × 10^7^ cells was collected after 48 h. EVs were purified by differential centrifugation and concentration as previously described [29]. Briefly, hMSC-Adi-conditioned medium (hAdi-CM) was centrifuged for 10 min at 2000 g to eliminate cell contamination. Supernatants were further centrifuged for 20 min at 3500 g, filtered through a 0.45 μm filter and concentrated thanks to Amicon Ultra-15 Centrifugal Filter Units with 10 kDa cut-off (Millipore). EVs were pelleted by ultracentrifugation at 110,000 g for 70 min. The EV pellets were either resuspended in DMEM, or lysed with Extract-all for RNA extraction or washed with PBS, pelleted again and resuspended in PBS.

### RNA expression measurement

#### RNA isolation

Total RNA was extracted using Extract-All reagent (Eurobio, Les Ulis, France), according to the manufacturer’s instructions. Total RNA was digested with DNase I (Roche Diagnostics, Meylan, France) and quantified by Nanodrop at 260 nm wavelength.

#### mRNAs expression analysis

Total RNA was reverse transcribed using Maxima First-Strand cDNA Synthesis kit (Fermentas, Thermoscientific, France), and subjected to quantitative real-time PCR on the LightCycler® Carousel-Based System (Roche Diagnostics) using the LightCycler® Fast Start DNA Master SYBR® Green I kit as described previously and specific primers designed using Oligo 6 software (MedProbe, Oslo, Norway) (Table 1) [16]. Results were normalized against expression of the indicated genes and compared to the expression level as baseline by the 2-ΔΔCt method, and log2-transformed fold changes of normalized 2-deltaCT.

**Table 1:** **mRNA primer sequences**

**mRNA**	**Primers**	**Accession numbers**
Human specific primers		
*hPPAR*γ	F: 5′-GCTTCTGGATTTCACTATGG-3′	NM_005037
	R: 5′-AAACCTGATGGCATTATGAG-3′	
*hLeptin*	F: 5′-ATTTCACACACGCAGTCAGT-3′	NM_00230
	R: 5′-GAAGAAGATCCCGGAGGT-3′	
*hCEBP*α	F: 5′-ACTGGGACCCTCAGCCTTG-3′	NM_004364
	R: 5′-TGGACTGATCGTGCTTCGTG-3′	
*hCEBP*δ	F: 5′-ACGACGACGAGAGCGCCATC-3′	NM_005195
	R: 5′-CGCCCGCCTTGTGATTGC-3′	
*hOC*	F: 5′-ATGAGAGCCCTCACACTCCTC-3′	NM_199173
	R: 5′-GCCGTAGAAGCGCCGATAGGC-3′	
*hOP*	F: 5′-CCTGCCAGCAACCGAAGTTT-3′	NM_001040058.1
	R: 5′-ACTGTCCTTCCCACGGCTGT-3′	
*hYWHAZ*	F: 5′-GGTCATCTTGGAGGGTCGTC-3′	NM_145690
	R: 5′-GTCATCACCAGCGGCAAC-3′	
*hGAPDH*	F: 5′-GTTCCAATATGATTCCACCC-3′	NM_002046.5
	R: 5′-AGGGATGATGTTCTGGAGAG-3′	
*h36B4*	F: 5′-CGACCTGGAAGTCCAACTAC-3′	NM_001002.3
	R: 5′-AGCAACATGTCCCTGATCTC-3′	
Mouse specific primers		
*mPPAR*γ	F: 5′-TTTTCAAGGGTGCCAGTTTC-3′	NM_001127330
	R: 5′-AATCCTTGGCCCTCTGAGAT-3′	
*mAdiponectin*	F: 5′-CCCAGTCATGCCGAAGA-3′	NM_009605.4
	R: 5′-TACATTGGGAACAGTGACGC-3′	
*mCEBP*α	F: 5′-TTACAACAGGCCAGGTTTCC-3′	NM_007678
	R: 5′-CTCTGGGATGGATCGATTGT-3′	
*mGAPDH*	F: 5′-GGCATTGCTCTCAATGACAA-3′	NM_008084
	R: 5′-TGTGAGGGAGATGCTCAGTG-3′	
*mActin*	F: 5′-AATTTCTGAATGGCCCAGGT-3′	NM_007393
	R: 5′-GGTAAGGTGTGCACTTTTATTGG-3′	

Shown are the primer sequences and Genbank accession numbers. F: forward; R: reverse. h: human; m: mouse. PPARγ, peroxisome proliferator-activated receptor gamma; CEBPα and δ, CCAAT/enhancer binding protein alpha and delta; YWHAZ, tyrosine 3-monooxygenase/tryptophan 5- monooxygenase activation protein, GAPDH, Glyceraldehyde 3-phosphate dehydrogenase. Human and mouse primers for PPARγ amplified isoforms 1 and 2.

For interspecies experiments, subsequent PCR products were migrated on 1.8% agarose gel for visualization.

#### miRNA expression analysis

Total RNA was reverse transcribed using the miScript II RT Kit (Qiagen), and subjected to quantitative real-time PCR on the LightCycler® Nano System (Roche Diagnotics) using the miScript SYBR Green PCR Kit (Qiagen) according to the manufacturer’s instructions. Specific primers are listed in Table 2. Results were normalized against RNU6P expression and compared to the expression level as baseline by the 2-ΔΔCt method, and log2-transformed fold changes of normalized 2-deltaCT.

**Table 2:** **miRNA primer sequences**

**miRNA**	**Primers**	**Reference or accession number**
*miR-30c*	5′-TGTAAACATCCTACACTCTCAGC-3′	[25]
*miR-138*	5′-GCTATTTCACGACACCAGGGTT-3′	NR_029680.1
*miR-125a*	5′-GGTCCCTGAGACCCTTTAACCT-3′	NR_029694.1
*miR-125b*	5′-TCCCTGAGACCCTAACTTGTGA-3′	NR_029693.1
*miR-31*	5′-GGAGGCAAGATGCTGGCATA-3′	NR_029505.1
*RNU6P*	5′-GTGCTCGCTTCGGCAGCACATAT-3′	NR_004394.1

Shown are the primer sequences and Genbank accession numbers.

### Electron microscopy of EVs

EVs from hMSC-Adi were labeled as described by Thery et al. [29]. Briefly, they were fixed in 2% paraformaldehyde in PBS, loaded onto formvar carbon-coated grids and incubated during 20 minutes. After extensive washing with PBS, EVs were postfixed in 1% glutaraldehyde for 5 min, extensively washed in distilled water and embedded in a mixture of uranyl acetate and methyl cellulose for negative coloration. They were then examined in a Zeiss 902, 80 kV transmission electron microscope.

### Statistical analysis

Results are expressed as median of experimental data, using quartiles [Q1;Q3] to evaluate variability around this value. All statistical comparisons were made using a non-parametric Mann–Whitney U-Test, using SPSS software version 18. Differences were considered significant at p < 0.05 (*p < 0.05, **p < 0.01).

## Abbreviations

Adi: Adipocyte
BM: Bone Marrow
CEBPα and δ: CCAAT/enhancer binding protein alpha and delta
CM: Conditioned Medium
EVs: Extracellular Vesicles
GAPDH: Glyceraldehyde 3-phosphate dehydrogenase
hMSC: Human Mesenchymal Stem Cell
miRNAs: microRNAs
OC: Osteocalcin
Ost: Osteoblast
OP: Osteopontin
PPARγ: Peroxisome proliferator-activated receptor gamma
RNAs: RiboNucleic Acids
RT-qPCR: Reverse Transcription-quantitative Polymerase Chain Reaction
YWHAZ: Tyrosine 3-monooxygenase/tryptophan 5- monooxygenase activation protein
